# *Mycobacterium abscessus* bacteremia complicated by sepsis and septic shock in a patient with multiple comorbidities: a case report

**DOI:** 10.3389/fimmu.2026.1834557

**Published:** 2026-05-08

**Authors:** Yuanyuan Xu, Lusheng Wang, Huihui Li, Pingping Zhao, Kaixuan Zhang, Sudi Zhu, Mengyu Zhang

**Affiliations:** 1 Laboratory Department, Wanbei Coal Electric Group General Hospital, Suzhou, Anhui, China; 2 Laboratory Department, Wanbei Coal Electric Group General Hospital Key Laboratory of Tuberculosis Detection of Suzhou City, Suzhou, Anhui, China; 3 Central Laboratory, Wanbei Coal Electric Group General Hospital, Suzhou, Anhui, China

**Keywords:** *Mycobacterium abscessus*, bacteraemia, sepsis, septic shock, case report

## Abstract

**Introduction:**

*Mycobacterium abscessus* is a rapidly growing nontuberculous mycobacterium that most commonly causes pulmonary, skin and soft tissue, or postoperative wound infections. Bloodstream infection due to *M. abscessus* is uncommon and is usually reported in patients with immune dysfunction, major comorbidity, or healthcare-associated exposure.

**Case presentation:**

We describe a 66-year-old man who presented with recurrent fever, myalgia, fatigue, dizziness, cough, and urinary urgency. His medical history was notable for poorly controlled type 2 diabetes mellitus, hypertension, prior cerebral infarction, coronary artery bypass grafting, lumbar spine surgery, and a recent episode of septic shock caused by *Mycobacterium abscessus*. During hospitalization, he developed recurrent high-grade fever and hemodynamic instability consistent with septic shock. Blood cultures yielded *M. abscessus*, while sputum smear microscopy demonstrated acid-fast bacilli; GeneXpert testing was negative for *Mycobacterium tuberculosis*. Species identification was established by MALDI-TOF mass spectrometry and further verified by melting-curve analysis. Additional evaluation showed persistent systemic inflammation, thrombocytopenia, cardiac dysfunction, and impaired cellular immunity. He received combination antimicrobial therapy together with supportive treatment, but remained intermittently febrile and symptomatic. He was discharged against medical advice on hospital day 4 at his family’s request.

**Conclusion:**

This case highlights that *M. abscessus* bacteremia may cause severe sepsis and septic shock in medically complex patients even in the absence of classical immunosuppressive therapy. Early clinical suspicion, culture-based microbiological confirmation, exclusion of tuberculosis, and timely individualized multidrug treatment are essential in patients with recurrent fever and poor response to conventional antibacterial therapy.

## 1 Introduction

*Mycobacterium abscessus* is a rapidly growing nontuberculous mycobacterium increasingly recognized as an important opportunistic pathogen because of its intrinsic antimicrobial resistance, environmental ubiquity, and association with healthcare-related infection (1–3). It most commonly causes pulmonary, skin and soft tissue, and postoperative or device-related infections, whereas bloodstream infection is uncommon. Available guidance and cohort data suggest that severe bloodstream infection due to rapidly growing nontuberculous mycobacteria occurs predominantly in patients with substantial comorbidity, immune dysfunction, invasive devices, or prior healthcare exposure, and that *M. abscessus* complex may be associated with greater antimicrobial resistance and poorer outcomes than other rapidly growing mycobacteria (4, 5).

The diagnosis of *M. abscessus* bacteremia is particularly challenging because its clinical presentation is non-specific and may initially mimic conventional bacterial sepsis, tuberculosis, or other common infectious syndromes (6, 7). Definitive diagnosis requires microbiological confirmation with culture and species-level identification, as acid-fast smear positivity alone cannot reliably distinguish nontuberculous mycobacteria from *Mycobacterium tuberculosis* (8). This distinction is particularly important in patients with recurrent fever and incomplete response to empirical antibacterial therapy, especially when prior healthcare exposure is present (9).

Here, we report a case of microbiologically confirmed *M. abscessus* bacteremia complicated by sepsis and septic shock in a 66-year-old man with poorly controlled diabetes mellitus, prior major surgery, structural heart disease, and evidence of impaired cellular immunity. This case is clinically instructive because it highlights the potential for severe invasive *M. abscessus* infection in the absence of classical immunosuppressive therapy and underscores the importance of early suspicion, definitive microbiological identification, and careful assessment of host vulnerability in patients with recurrent fever and poor response to standard antimicrobial treatment.

## 2 Case presentation

A 66-year-old man from Anhui Province, China, was admitted to our hospital with a 10-day history of recurrent fever. The illness had begun without an obvious precipitating factor and was accompanied by generalized myalgia, fatigue, dizziness, occasional non-productive cough, and urinary urgency. He denied chills, headache, sputum production, abdominal pain, diarrhea, dysuria, jaundice, pruritus, or bleeding manifestations. Empirical intravenous azithromycin and amikacin administered at a local clinic resulted in transient defervescence; however, the fever recurred after treatment was discontinued, prompting further evaluation. Throughout this period, he remained alert and had no focal neurological deficits. Appetite and sleep were relatively preserved, and there was no reported weight loss.

His medical history was notable for several chronic conditions, including hypertension of 3 years’ duration, previous cerebral infarction without residual neurological sequelae, and type 2 diabetes mellitus with suboptimal glycemic control (HbA1c 8.1%) treated with empagliflozin. He had undergone coronary and cerebral angiography followed by coronary artery bypass grafting in February 2023, and lumbar discectomy with laminectomy for spinal stenosis in April 2024. Importantly, he had experienced septic shock and sepsis caused by *M. abscessus* approximately 2 months before the current admission, and blood culture at that time had likewise yielded *M. abscessus*. This raised concern for recurrent or persistent bloodstream infection rather than an isolated single episode. Additional history included cervical spondylosis and prior blood transfusion. He denied smoking, alcohol consumption, tuberculosis, viral hepatitis, chronic bronchitis, nephritis, recent travel, or known infectious exposure.

On admission, his temperature was 36.5 °C, blood pressure 127/89 mmHg, heart rate 95 beats/min, respiratory rate 19 breaths/min, and oxygen saturation 99% on room air. He was alert and cooperative but appeared malnourished. Physical examination revealed no jaundice, rash, petechiae, superficial lymphadenopathy, hepatosplenomegaly, or peripheral edema. Breath sounds were clear bilaterally, and cardiac auscultation demonstrated a regular rhythm without murmurs. A well-healed median sternotomy scar and a surgical scar over the right lower extremity were noted. Abdominal and neurological examinations were otherwise unremarkable.

Soon after admission, the patient developed recurrent high-grade fever, with a peak temperature of 39.2 °C, accompanied by persistent myalgia, fatigue, dizziness, cough, urinary urgency, chest tightness, and nausea. During hospitalization, he became hemodynamically unstable, with hypotension reaching 81/62 mmHg, consistent with septic shock. Subsequent bedside monitoring showed a heart rate of 106 beats/min, blood pressure of 106/67 mmHg, and oxygen saturation of 100% under supplemental oxygen.

**Figure 1:**
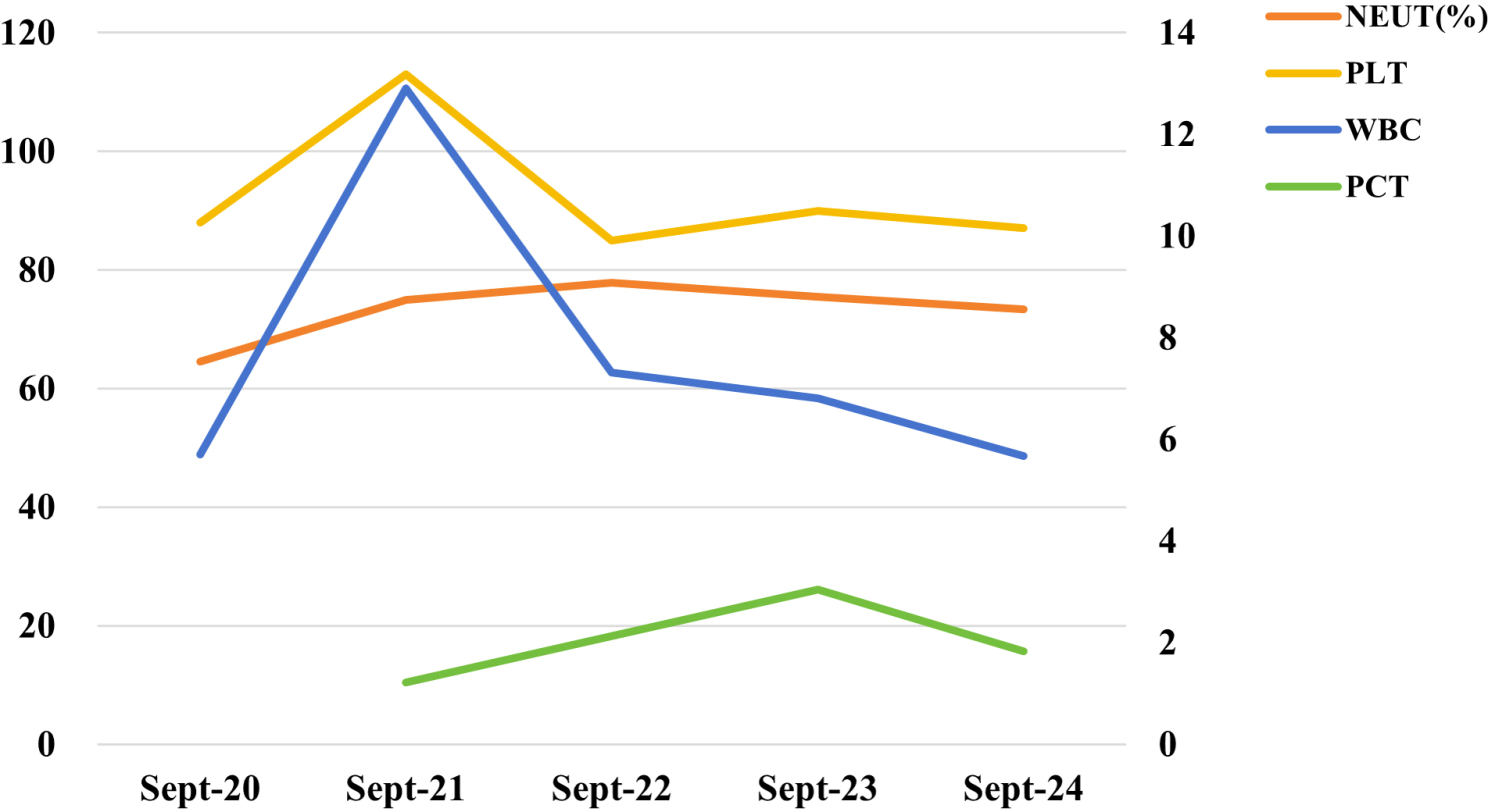
Dynamic changes in infection-related biomarkers, including WBC count, neutrophil percentage, platelet count, and procalcitonin during hospitalization.

Laboratory evaluation showed persistent systemic inflammation and multisystem involvement. The white blood cell count rose from 5.70 × 10^9/L on September 20 to 12.90 × 10^9/L on September 21, then gradually declined to 5.67 × 10^9/L by September 24. Neutrophil proportions remained consistently elevated (64.5%–77.8%), whereas platelet counts were persistently reduced (85–113 × 10^9/L). Procalcitonin levels were elevated and fluctuated during hospitalization, peaking at 3.051 ng/mL on September 23; serum amyloid A was markedly increased at 251.47 mg/L. Additional investigations showed low-normal total lymphocyte count with abnormalities in lymphocyte subsets, mild hepatic dysfunction, elevated blood urea nitrogen, hyponatremia, and hypertriglyceridemia. Urinalysis demonstrated marked glycosuria with leukocyturia, bacteriuria, and funguria. Serological testing for influenza virus, hepatitis C virus, syphilis, HIV, TORCH, and autoimmune antibodies was negative, and Brucella agglutination testing was likewise negative. Dynamic changes in infection-related biomarkers during hospitalization are summarized in **Figure 1** and **Table 1**. As shown in **Figure 1**, the biomarker profile was characterized by transient leukocytosis, persistently elevated neutrophil proportions, fluctuating but increased procalcitonin levels, and sustained thrombocytopenia. Together, these abnormalities were consistent with persistent systemic inflammatory activation and substantial infection burden in the setting of severe disease.

**Table 1:** Dynamic changes in infection-related biomarkers.

Parameter	Sept-20	Sept-21	Sept-22	Sept-23	Sept-24
WBC	5.7	12.9	7.31	6.8	5.67
NEUT (%)	64.50	74.9	77.8	75.5	73.4
PLT	88	113	85	90	87
PCT		1.218		3.051	1.831

**Figure 2:**
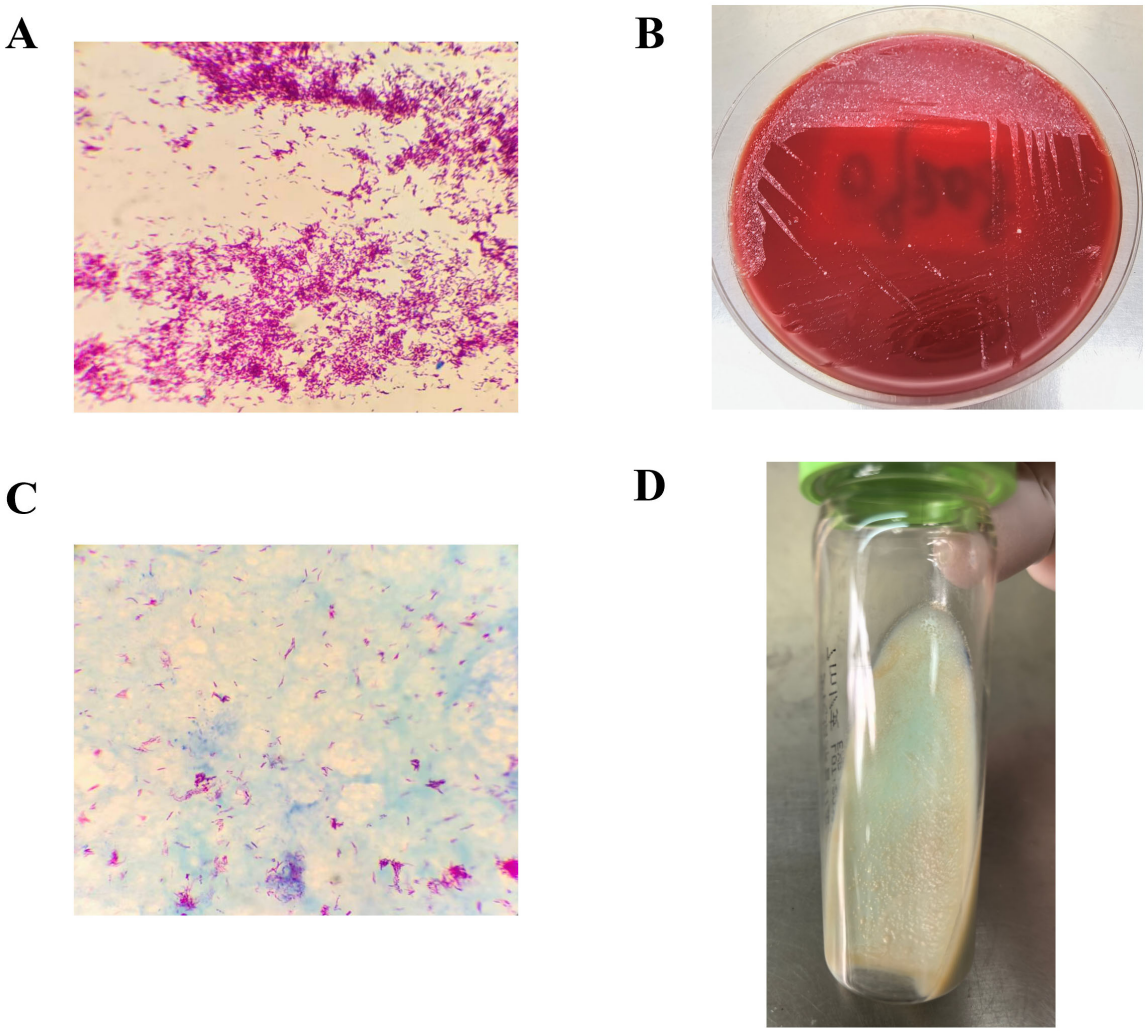
Microbiological identification of Mycobacterium abscessus from blood culture. **(A)** Gram staining of the positive blood culture smear using the Zhuhai Baso Gram stain kit demonstrated Gram-positive bacilli; **(B)** After 72 h of incubation, pinpoint colonies were observed on Columbia blood agar plates; **(C)** Acid-fast staining using the Zhuhai Baso acid-fast stain kit showed acid-fast-positive bacilli. **(D)** After 1 week of incubation on Zhuhai Baso Löwenstein–Jensen medium, yellow, rough, small colonies were observed.

Microbiological studies were pivotal to diagnosis. Blood cultures yielded *M. abscessus*. Acid-fast staining demonstrated acid-fast bacilli, and sputum smear microscopy showed 8 acid-fast bacilli per 300 microscopic fields. By contrast, rapid molecular testing of sputum using GeneXpert was negative for Mycobacterium tuberculosis complex DNA and rifampicin resistance-associated rpoB mutations, arguing against pulmonary tuberculosis. Characteristic colony morphology was observed on blood agar and Löwenstein–Jensen media. The isolate was identified as *M. abscessus* by MALDI-TOF mass spectrometry (Bruker, Germany) and further verified by melting-curve analysis (Zhishan platform, Xiamen, China). Sputum culture also showed heavy growth of Candida albicans. Representative microbiological findings from the positive blood culture are shown in **Figure 2**, illustrating the sequential laboratory evidence that supported identification of *M. abscessus*.

Because of the severity of infection in the absence of classical immunosuppressive therapy, peripheral blood immune evaluation was performed on 20 September 2024 as part of routine clinical assessment. Blood samples were collected on 20 September 2024. Lymphocyte subset analysis was performed by 10-color flow cytometry on a BECKMAN COULTER instrument, and NK-cell functional assessment was performed by 6-color flow cytometry on an Agilent instrument according to the manufacturers’ instructions. Immune measurements were obtained at a single time point only, and repeated testing was not available. Autoimmune serologies, including ANA and extractable nuclear antigen-related antibodies, were negative or within the normal range. Immunological evaluation showed reduced T-cell, CD8^+ T-cell, B-cell, and NK-cell absolute counts, together with markedly decreased IFN-γ- and granzyme B-related functional indices in cytotoxic lymphocyte subsets, supporting clinically meaningful impairment of cellular antimycobacterial immunity (**Table 2**).

**Table 2:** Key immunological findings during hospitalization.

Parameter	Result	Reference range	Interpretation
Total lymphocyte count	1010/μL	790–4210	Low-normal
CD3^+ T-cell absolute count	914/μL	955–2860	Decreased
CD8^+ T-cell absolute count	236/μL	320–1250	Decreased
B-cell absolute count	43/μL	90–580	Decreased
NK-cell absolute count	62/μL	150–900	Decreased
CTL absolute count	80/μL	258–958	Decreased
GzmB^+ CD8^+ T-cell absolute count	59/μL	71–541	Decreased
IFN-γ^+ CD8^+ T-cell absolute count	9/μL	91–445	Markedly decreased
IFN-γ^+ NK-cell absolute count	3/μL	34–245	Markedly decreased
NK-cell absolute count (functional panel)	30/μL	109–780	Markedly decreased

Imaging studies revealed extensive cardiopulmonary and systemic abnormalities. Chest computed tomography showed bilateral inflammatory changes, emphysema, patchy right lung opacities, cardiomegaly, localized pericardial thickening, coronary artery calcification, postoperative thoracic changes, and a nodular shadow along the anterior margin of the ascending aorta; cholelithiasis was also noted. Transthoracic echocardiography demonstrated postoperative changes after CABG, segmental left ventricular wall motion abnormalities with a left ventricular apical aneurysm, left heart enlargement, reduced left ventricular systolic function, dilatation of the aortic root and ascending aorta, mild aortic regurgitation, moderate mitral regurgitation, mild-to-moderate tricuspid regurgitation, and mild pulmonary hypertension. Cardiac biomarkers were elevated, including troponin I at 0.192 μg/L and NT-proBNP at 7720 pg/mL, indicating substantial cardiac stress in the setting of severe systemic infection. Additional ultrasonography showed diffuse thyroid disease, cholelithiasis with splenomegaly, and prostatic enlargement with calcification. Electrocardiography demonstrated atrial tachycardia, abnormal Q waves in the inferior and anterolateral leads, and ST–T abnormalities.

Taken together, the recurrent febrile illness, previous history of *M. abscessus* sepsis, positive blood cultures for *M. abscessus*, acid-fast smear positivity, exclusion of tuberculosis, and progressive hemodynamic compromise supported a diagnosis of *M. abscessus* bacteremia complicated by sepsis and septic shock. Concomitant diagnoses included type 2 diabetes mellitus, unstable angina, post-CABG status, postoperative lumbar spine disease, coagulopathy, hypertriglyceridemia, hyponatremia, atrial tachycardia, senile valvular heart disease, benign prostatic hyperplasia with calcification, cholelithiasis, hypersplenism, left ventricular apical aneurysm, and left heart enlargement.

The patient was treated with azithromycin, amikacin, rifampicin, and clarithromycin, together with esomeprazole, ibuprofen, lysine aspirin, insulin, nitroglycerin, dopamine, intravenous fluids, and other supportive measures for fever control, glycemic management, gastric protection, and hemodynamic stabilization. Multidisciplinary consultations involving cardiology, cardiovascular surgery, and urology were obtained, and conservative management of the underlying comorbidities was continued. Despite these interventions, he remained intermittently febrile and continued to report systemic symptoms, suggesting incomplete control of infection. On hospital day 4, his family requested discharge against medical advice. He was discharged after signing the relevant documentation and was advised to continue anti-infective and supportive treatment with close outpatient follow-up. Further follow-up data were unavailable.

## 3 Discussion

*M. abscessus* is a rapidly growing nontuberculous mycobacterium increasingly recognized as a clinically important opportunistic pathogen because of its intrinsic antimicrobial resistance, association with healthcare-related exposure, and capacity to cause chronic, invasive, or disseminated disease (10, 11). In routine practice, it most commonly presents as pulmonary, skin and soft tissue, or postoperative wound infection, whereas bloodstream infection remains distinctly uncommon. Available cohort data suggest that rapidly growing mycobacterial bloodstream infections are associated with substantial morbidity, and that *M. abscessus* complex, in particular, carries a more resistant microbiological profile and poorer clinical outcomes than bloodstream infections caused by other rapidly growing mycobacteria (12, 13). Representative published reports of *M. abscessus* bacteremia are summarized in **Table 3**, highlighting recurrent predisposing themes such as prior major surgery, healthcare-associated exposure, immune dysfunction, and poor clinical outcome.

**Table 3:** Selected representative reports of *Mycobacterium abscessus* bacteremia.

Author, year	Clinical setting	Key finding	Relevance to the present case
Sarma et al. (24), 2011	Post-CABG patient	Bacteremia after cardiac surgery.	Highlights prior major cardiac surgery as a marker of healthcare exposure rather than a proven causal source.
Liu et al. (25), 2013	Intravenous infusion exposure	Bacteremia may present with septic shock.	Supports the severe systemic presentation seen in our patient.
Fukui et al. (18), 2015	Disseminated infection in an immunosuppressed host	Blood culture positivity associated with severe disseminated disease.	Supports the prognostic significance of bacteremia.
Comba et al. (12), 2021	RGM bloodstream infection cohort	*M. abscessus* complex showed higher resistance and worse outcomes.	Provides cohort-level evidence for poor prognosis.
Wu et al. (26), 2024	Idiopathic CD4 lymphocytopenia	Disseminated infection with bacteremia in cellular immune impairment.	Supports the role of impaired cellular immunity.
Vithiya et al. (27), 2024	Cardiac intervention-related endocarditis	Repeated blood culture positivity was key to diagnosis.	Relevant to structural heart disease and prior cardiac procedures.
Philip et al. (28), 2025	NTM bloodstream infection cohort	Diabetes and invasive exposure were common; *M. abscessus* complex had the worst prognosis.	Strong recent evidence supporting the high-risk profile of the present case.

Several aspects make the present case noteworthy. Most importantly, the patient had microbiologically confirmed *M. abscessus* bacteremia complicated by sepsis and septic shock, indicating true invasive disease rather than colonization or limited local infection. In addition, he had experienced *M. abscessus*-associated septic shock only two months earlier, and blood culture at that time had also been positive for *M. abscessus*, raising the possibility of recurrent or persistent bloodstream infection. A further distinctive feature was the coexistence of multiple host vulnerabilities, including poorly controlled diabetes mellitus, poor nutritional status, prior major surgery, structural heart disease, and evidence of impaired cellular immunity. Taken together, these features help explain the severity of infection in this patient.

This case also illustrates the likely interplay between host susceptibility and healthcare-associated exposure. Previous reports have described *M. abscessus* bacteremia in patients with indwelling catheters, contaminated infusates, cardiac procedures, stem cell transplantation, and prior major surgery. In the present case, the source of bloodstream infection could not be definitively established, but a healthcare-associated origin remains biologically plausible. The patient had a history of major prior procedures, including coronary artery bypass grafting and lumbar spine surgery, which should be interpreted primarily as markers of antecedent healthcare exposure rather than evidence of a direct causal relationship with the current infection. These procedures may also represent possible contexts for occult postoperative or procedure-related infection, although no direct source was identified. In addition, endovascular involvement could not be fully excluded, given his substantial structural cardiac disease, prior CABG, left ventricular apical aneurysm, and significant cardiac dysfunction, although no definite vegetation was identified on transthoracic echocardiography. Likewise, while no catheter-related source was documented, repeated healthcare exposure and prior invasive management increase the plausibility of an unrecognized healthcare-associated bloodstream source. Overall, this case highlights the importance of careful evaluation for occult postoperative, catheter-related, or endovascular foci when *M. abscessus* bacteremia is identified in medically complex patients, while avoiding overinterpretation of prior surgery as proof of causality.

The diagnosis was clinically challenging because the presenting features were non-specific. Recurrent fever, myalgia, fatigue, cough, thrombocytopenia, and elevated inflammatory markers can easily be attributed to routine bacterial sepsis, pulmonary infection, urinary infection, or even non-infectious inflammatory conditions. In this patient, the diagnosis was supported by a coherent microbiological framework: positive blood culture for *M. abscessus*, acid-fast bacilli on sputum smear, negative GeneXpert testing for *Mycobacterium tuberculosis*, and species-level identification by MALDI-TOF mass spectrometry with melting-curve confirmation. This point is clinically important, because acid-fast smear positivity does not distinguish tuberculosis from NTM infection; definitive diagnosis depends on culture and reliable species identification (14, 15).

The laboratory profile also supports the severity of systemic infection in this patient. As illustrated in **Figure 1**, procalcitonin remained elevated during hospitalization and platelet counts stayed persistently reduced, while neutrophil predominance was maintained throughout the clinical course. Procalcitonin remained elevated during hospitalization and peaked at 3.051 ng/mL, while serum amyloid A was markedly increased, consistent with persistent systemic inflammatory activation. At the same time, platelet counts remained persistently low, suggesting thrombocytopenia in the setting of severe infection and possible sepsis-associated hematological disturbance. Together with the patient’s hemodynamic instability, these biomarker trends further support the clinical assessment of sepsis and septic shock. Beyond microbiological confirmation, the present case is also notable for the pattern of host immune dysfunction identified during hospitalization.

Another important feature of this case is the presence of immune dysfunction despite the absence of classical immunosuppressive therapy. The patient had reduced lymphocyte, NK-cell, total T-cell, and CD8^+ T-cell counts, together with low IFN-γ- and granzyme B-related functional indices, suggesting clinically relevant impairment of cellular antimycobacterial immunity. This finding is immunologically important because host defense against nontuberculous mycobacteria depends largely on intact cell-mediated immunity, particularly IFN-γ-dependent signaling, which is essential for macrophage activation and intracellular control of mycobacteria (16). In this context, the reduced IFN-γ-related indices observed in our patient may reflect impaired antimycobacterial effector signaling and diminished containment of *M. abscessus* after bloodstream invasion. Likewise, the reduction in NK-cell and CD8^+ T-cell compartments provides a biologically plausible explanation for impaired early containment of infection and progression to sepsis and septic shock, while the low granzyme B-related indices further support reduced cytotoxic immune competence rather than isolated numerical lymphopenia (17).

Importantly, these abnormalities are unlikely to be explained by classical immunosuppression, as the patient had not received chemotherapy, transplantation-related therapy, or long-term immunosuppressive agents. Instead, the overall pattern appears more consistent with functional immune impairment in the setting of advanced age, poorly controlled diabetes, malnutrition, recurrent severe infection, and chronic multisystem disease. Previous reports of disseminated or bacteremic *M. abscessus* infection have mainly involved patients with overt immunocompromise (18, 19). By contrast, our patient did not fit these conventional categories, yet still demonstrated clinically meaningful immune vulnerability. This distinction is important because it suggests that severe *M. abscessus* bacteremia may also occur in patients with non-classical but biologically significant defects in antimycobacterial host defense.

The cardiovascular context further increased the complexity of this case. The patient had post-CABG status, left ventricular apical aneurysm, left heart enlargement, reduced left ventricular systolic function, valvular regurgitation, atrial tachycardia, and markedly elevated NT-proBNP and troponin I levels, indicating limited cardiovascular reserve at the time of severe systemic infection. Deep-seated endovascular involvement could not be fully excluded, although no definite vegetation or other clear source was documented. Even so, the combination of bacteremia, septic shock, and significant structural heart disease likely contributed to the severity of the short-term clinical course.

Management of *M. abscessus* infection remains challenging because of intrinsic resistance to multiple antimicrobial agents, variable macrolide susceptibility, inducible resistance mechanisms such as *erm(41)*, and the frequent need for source control (20, 21). Although no dedicated guideline exists specifically for *M. abscessus* bacteremia, current ATS/ERS/ESCMID/IDSA guidance for *M. abscessus* pulmonary disease provides the closest therapeutic framework and emphasizes susceptibility-based treatment for macrolides and amikacin, identification of inducible or mutational macrolide resistance, and the use of an initial multidrug regimen containing at least three active agents whenever possible. In strains with inducible or mutational macrolide resistance, macrolides may still be retained for potential immunomodulatory effects but should not be counted as active drugs.

In the present case, antimicrobial susceptibility testing, subspecies-level identification, and molecular resistance profiling, including *erm(41)* analysis, were not available. This represents a major limitation, because the microbiological adequacy of the reported regimen and the likely resistance profile of the isolate could not be fully assessed. In the present case, antimicrobial susceptibility testing, subspecies-level identification, and molecular resistance profiling, including *erm(41)* analysis, were not available. This represents a major limitation, because the microbiological adequacy of the reported regimen and the likely resistance profile of the isolate could not be fully assessed. The patient received azithromycin, amikacin, rifampicin, and clarithromycin together with intensive supportive therapy, reflecting the actual clinical management administered during hospitalization rather than an optimized susceptibility-guided regimen. We further acknowledge that the simultaneous use of azithromycin and clarithromycin is difficult to justify within current therapeutic frameworks for *M. abscessus* infection, as concurrent use of two macrolides is not part of standard susceptibility-guided treatment. Likewise, rifampicin is generally considered to have limited and unreliable activity against *M. abscessus* and would not usually be regarded as a preferred core agent in a modern multidrug regimen. Accordingly, this antimicrobial combination should not be interpreted as evidence-based preferred therapy for *M. abscessus* bacteremia, but rather as a real-world empiric approach used in a medically fragile patient with severe infection, rapid clinical deterioration, and incomplete microbiological characterization.

Persistent fever and systemic symptoms during hospitalization suggested incomplete infection control. In the absence of susceptibility data, it remains difficult to determine whether this reflected intrinsic resistance, inducible macrolide resistance, suboptimal regimen composition, incomplete source control, or the combined effects of severe host vulnerability and early treatment interruption. This limitation reflects a common real-world challenge in severe NTM infection, particularly when comprehensive microbiological characterization cannot be completed before clinical deterioration or premature discharge.

This report has several additional limitations. Antimicrobial susceptibility testing, subspecies-level identification, and molecular resistance analysis, including assessment of *erm(41)*-associated inducible macrolide resistance, were not available, limiting evaluation of the appropriateness of the antimicrobial regimen and microbiological interpretation of treatment response. Repeated blood culture documentation and time-to-positivity data were also unavailable, making it difficult to further characterize the microbiological dynamics of the bloodstream infection. The precise infectious source could not be established, and no definitive evidence of catheter-related infection, endovascular infection, or other deep-seated focus was obtained. In addition, because the patient was discharged against medical advice on hospital day 4, no further follow-up data were available. As a result, the final treatment response, microbiological clearance, and long-term clinical outcome could not be determined, which limits assessment of both prognosis and therapeutic efficacy. These limitations should be considered when interpreting the clinical course and generalizing the findings.

This case also has diagnostic implications. In regions where tuberculosis remains prevalent, a positive acid-fast smear may prompt presumptive treatment for *Mycobacterium tuberculosis* (22). Similarly, the heavy growth of Candida albicans in sputum was considered more likely to represent colonization than the primary cause of systemic illness. This interpretation is supported by the fact that sputum is a non-sterile specimen, while there was no corresponding clinical, radiological, or microbiological evidence of invasive fungal infection. Although concomitant airway colonization or a minor contributory role cannot be completely excluded, the available evidence strongly favored *M. abscessus*, which was directly identified from blood culture, as the clinically relevant pathogen driving sepsis and septic shock. These findings underscore the importance of cautious interpretation of non-sterile-site microbiology and of prioritizing bloodstream evidence when determining the principal infectious diagnosis.

From a broader perspective, this case adds to the limited literature on *M. abscessus* bacteremia in medically complex adults. Published studies suggest that *M. abscessus* complex bloodstream infection is associated with particularly poor outcomes among rapidly growing mycobacterial bloodstream infections, especially in patients with diabetes, immune dysfunction, invasive devices, or prior surgical exposure (12, 23). The present case extends this literature by showing that recurrent M. abscessus bacteremia with septic shock may occur in an elderly patient without classical immunosuppressive therapy but with major comorbidity, functional cellular immune impairment, and extensive prior healthcare exposure.

In summary, this case highlights that *M. abscessus* bacteremia should be considered in patients with recurrent fever, poor response to conventional antibacterial therapy, prior healthcare exposure, and acid-fast microbiological findings. Early recognition, culture-based confirmation, species-level identification, and individualized multidrug treatment are essential. Equally important, this case underscores that functional immune impairment and complex medical comorbidity may be sufficient to predispose to severe invasive *M. abscessus* infection, even in the absence of overt immunosuppression.

## 4 Conclusion

This case documents a rare but clinically significant episode of *M. abscessus* bacteremia complicated by sepsis and septic shock in an elderly patient with diabetes, prior major surgery, structural cardiac disease, and evidence of impaired cellular immunity. It illustrates that invasive *M. abscessus* bloodstream infection may develop even in the absence of classical immunosuppressive therapy and may initially present with non-specific manifestations, thereby delaying diagnosis. The case underscores the importance of early clinical suspicion, prompt culture-based confirmation, reliable species-level identification, and timely individualized multidrug therapy. Clinicians should maintain particular vigilance for this diagnosis in medically complex patients with recurrent fever, prior healthcare exposure, and an unsatisfactory response to conventional antibacterial treatment.

## Data Availability

The original contributions presented in the study are included in the article/supplementary material. Further inquiries can be directed to the corresponding author.
